# Editorial: Model organisms in plant developmental biology—their effectiveness and limitations

**DOI:** 10.3389/fpls.2024.1478483

**Published:** 2024-09-13

**Authors:** Verónica S. Di Stilio, Neelima R. Sinha

**Affiliations:** ^1^ Department of Biology, University of Washington, Seattle, WA, United States; ^2^ Department of Plant Biology, University of California, Davis, CA, United States

**Keywords:** model organisms, model clade, plant development, Evo-Devo, #collectionseries

Model organisms represent an invaluable resource for fundamental and applied research allowing the identification of the mechanistic basis of evolutionary innovations. This Research Topic showcases studies performed on established and emerging model organisms in Plant Developmental Biology that have broad significance to the field. Increased phylogenetic breadth and availability of genomes and transgenic techniques have fostered innovative ideas and syntheses spanning the range from fossil analyses to single-cell sequencing. However, broad taxonomic applicability of the knowledge gained from studies on model organisms and relevance to the field of Evolutionary Developmental Biology (Evo-Devo) often remains unresolved.

To address such questions, this Research Topic focuses on new insights, latest discoveries, current challenges, and future perspectives on the use of model organisms and the extent to which the knowledge gained from them can be extrapolated. Authors were encouraged to identify the greatest unifying concepts in their sub-disciplines, as well as to put forward potential solutions to address the challenges emerging from the use of model plants.

## Core eudicots

There is still much to be learned in the classic core eudicot model *Arabidopsis thaliana*, such as description of a novel signaling pathway used by plant elicitor peptides to regulate root hair development (Jing et al.). *Cleome violacea* (Cleomaceae), in the sister lineage to Brassicaceae, utilizes comparisons to *Arabidopsis* to illuminate conserved genetic pathways in the evolution of nectary development suggesting a unique origin of nectaries in these sister lineages, while adding a reference point to only four other core eudicots with functional data on this aspect of flower development (Carey et al.).

*Economically significant models*: In the dicot model crop cotton (*Gossypium hirsutum*, Malvaceae), novel insights were gained on the role of the floral E class genes, the MADS box transcription factors *SEPALLATA*. Via targeted gene silencing combined with heterologous overexpression in *Arabidopsis*, the authors find that the cotton orthologs GhSEP interact with regulators of the transition to flowering, such as GhAP1 and GhLFY, leading them to propose a model of tetramer interaction in leaves, meristems, and floral organs (Chen et al.). In the model tree poplar (Salicaceae), Li et al. demonstrate that two BLADE ON PETIOLE orthologs function in secondary growth affecting wood production via heterologous overexpression in *Arabidopsis*.

The widespread stem parasitic plants in the *Cuscuta* genus (Convolvulaceae) offer a unique model to investigate this divergent plant growth strategy that results in enormous loss in agricultural fields. The authors present the advantages of using *Cuscuta* species as model organisms to illuminate the haustorium formation process using unique features such as self, hyper-, and cross-organ parasitism (Jhu and Sinha).

In the monocot model crop maize, the ubiquitous developmental regulators GF14 emerged as candidates for plant height from gene regulatory network analyses. Heterologous testing via overexpression in *Arabidopsis* resulted in decreased plant height providing further evidence that these genes are involved in regulating plant growth that directly impacts crop yield (Wang et al.).

## Early-diverging eudicots (Ranunculales)

The order Ranunculales occupies an interesting phylogenetic position as an early-diverging eudicot and sister group to the core eudicots. Four representatives of this order, encompassing two families, are included in this Research Topic.

The iconic state flower California poppy (*Eschscholzia californica*, Papaveraceae) is an emerging model with a genome in the making and the ability to conduct transient gene silencing. California poppy promises to help integrate traditional enquiries into the regulation of morphogenesis with emerging interest in secondary metabolites due to its abundance of benzylisoquinoline alkaloids in floral organs (Becker et al.).

*Thalictrum thalictroides* (Ranunculaceae) is amenable to high-efficiency targeted gene silencing. Combined with gene duplication from multiple rounds of polyploidy in the genus and expression analyses, the authors demonstrate the multiple roles of the MYB transcription factor family *MIXTA-like* and bridge the gap to reconstruct its ancestral function. In addition to its previously demonstrated role in papillate cells on the floral perianth, the authors demonstrate its involvement in leaf trichome development in *T. thalictroides*. Heterologous overexpression in tobacco demonstrates a conserved trichome function, supporting the dual role in the eudicot ancestor, presumably later parsed into distinct paralogs (Zahid et al.).

Damerval et al. characterize the land plant-specific TCP family of transcription factors in *Nigella damascena* (Ranunculaceae). On the one hand, the authors identify six clades of Class I TCP genes in *Nigella* tracing back to the origin of angiosperms, and three clades of Class II TCP with mostly redundant expression. On the other hand, the CYC/TB1 lineage are expressed distinctly suggesting more specific roles.

Sharma et al. review the unique ways in which the *Delphinieae* tribe, another Ranunculaceae model clade, contributes to understanding of the evolution, ecology, and development of complex flower symmetry involving a “hyperorgan” of synorganized spurred petaloid organs.

## Gymnosperms

In non-flowering seed plants (gymnosperms), where very few model systems have been developed, Tang et al. characterize a *Ginkgo biloba* variety with a range of abnormal leaf shapes from needle- to trumpet- type. The authors find anatomical and genetic evidence for changes in leaf polarity via shifts in the adaxial (dorsal) and abaxial (ventral) domains in combination with boundary defects resulting in tissue fusion.

## Bryophytes

Two articles highlight representatives of the non-vascular bryophytes, a liverwort and a hornwort. Singh and Bowman introduce us to the enigmatic *Ricciocarpos natans*, a liverwort that has secondarily evolved to be aquatic and monoicous from ancestral dioicy with sex chromosomes. *R. natans* thus provides an excellent platform to investigate the genomic consequences of sexual system and life habit transitions while also broadening the land plant perspective. Frangedakis et al. provide a valuable comparison of the hornwort *Anthoceros agrestis* to other model land plants while illustrating the unique features whose evolution it is well positioned to address, such as polyplastidy and symbiotic associations with fungi and cyanobacteria.

In closing, model organisms continue to be developed across land plants enriching the landscape for Evo-Devo studies. Here, we compiled contributions encompassing two non-vascular plants, a gymnosperm and ten angiosperms, comprising one monocot, four early-diverging eudicots, and five core eudicots. This sampling of model systems illustrates the possibilities for extending the frontiers of plant evolution and development, as well as pointing out current limitations.

